# Women's reproductive traits and cerebral small-vessel disease: A two-sample Mendelian randomization study

**DOI:** 10.3389/fneur.2023.1064081

**Published:** 2023-03-30

**Authors:** Zhenqian Wang, Jiawen Lu, Weipin Weng, Jie Zhang

**Affiliations:** ^1^School of Public Health (Shenzhen), Sun Yat-sen University, Shenzhen, Guangdong, China; ^2^Department of Neurology, The Second Xiangya Hospital, Central South University, Changsha, Hunan, China

**Keywords:** women's reproductive traits, cerebral small vessel disease measures, small vessel ischemic stroke, intracerebral hemorrhage, MRI markers, Mendelian randomization

## Abstract

**Background:**

Observational studies have suggested that women's reproductive factors (age at menarche (AAM), age at first birth (AFB), age at first sexual intercourse (AFS), age at natural menopause (ANM), and pregnancy loss) may influence the risk of cerebral small-vessel disease (CSVD) although the causality remains unclear.

**Methods:**

We conducted two-sample univariable Mendelian randomization (UVMR) and multivariable MR (MVMR) to simultaneously investigate the causal relationships between five women's reproductive traits and CSVD clinical [intracerebral hemorrhage (ICH) by location or small-vessel ischemic stroke (SVS)] and subclinical measures [white matter hyperintensities (WMH), fractional anisotropy (FA), and mean diffusivity (MD)], utilizing data from large-scale genome-wide association studies of European ancestry. For both UVMR and MVMR, the inverse-variance-weighted (IVW) estimates were reported as the main results. The MR-Egger, weighted median, generalized summary-data-based MR (GSMR), and MR-pleiotropy residual sum and outlier (MR-PRESSO) methods for UVMR and MVMR-Egger, and the MVMR-robust methods for MVMR were used as sensitivity analyses. Sex-combined instruments for AFS and AFB were used to assess the impact of sex instrumental heterogeneity. Positive control analysis was implemented to measure the efficacy of selected genetic instruments.

**Results:**

We found no evidence to support causal associations between genetic liability for women's reproductive factors and the risk of CSVD in UVMR (all *P*-values > 0.05). Using MVMR, the results were consistent with the findings of UVMR after accounting for body mass index and educational attainment (all *P*-values > 0.05). Sensitivity analyses also provided consistent results. The putative positive causality was observed between AAM, ANM, and ovarian cancer, ensuring the efficacy of selected genetic instruments.

**Conclusion:**

Our findings do not convincingly support a causal effect of women's reproductive factors on CSVD. Future studies are warranted to investigate specific estrogen-related physiological changes in women, which may inform current researchers on the causal mechanisms involved in cerebral small-vessel disease progression.

## Introduction

Globally, cerebral small-vessel disease (CSVD) is a major subtype of vascular cognitive impairment that has been alarmingly reported as an important precursor of dementia and full-blown strokes (1). It is the attributable cause of most spontaneous intracranial hemorrhage (ICH) and ~25% of ischemic strokes [known as small-vessel strokes (SVS)] (2). In general, CSVD can appear on structural magnetic resonance imaging (MRI) as white matter hyperintensities (WMH) of presumed vascular origin (3). Meanwhile, mounting evidence implies that the WMH burden is worse among women (4, 5), highlighting, in particular, that the effects of women's reproductive factors may be long-lasting and potentially influence brain trajectories later in life (6).

Female reproductive factors may play a key sex-specific role in CSVD, which could be attributed to the effect of estrogen on cerebrovascular function. The first menstrual period (menarche) is the most definitive sign of puberty and signals the beginning of the capacity to reproduce in women. The rise in gonadotrophins during puberty stimulates the ovaries to produce estradiol, triggering a series of dramatic physical, emotional, cognitive, and social changes in women. Such puberty hormonal events subsequently compose neural circuits for adult reproductive behaviors and regulate neuronal morphology, quantity, and function (7–9). During a woman's reproductive life, circulating levels of estrogen fluctuate during the menstrual cycle, and their concentration also changes in response to major reproductive events. Growing lines of evidence indicate that estrogen prevents endothelial dysfunction and atherosclerosis by promoting endothelial healing and increasing angiogenesis, thereby protecting against cardiovascular diseases (CVD) (10). However, the benefits of estrogen on cholesterol metabolism and endothelial function diminish as estrogen levels decline after menopause, resulting in postmenopausal women having more WMH than premenopausal women or men of the same age (11, 12). Similarly, a systematic review and meta-analysis found that women with a shorter reproductive lifespan, proxying for shorter estrogen exposure time, have a higher risk of CVD events, with a pooled relative risk of 1.31 for stroke (13). Collectively, estrogen exposure during a woman's lifetime is correlated with multiple reproductive factors, including menarche, first sex, pregnancy, and menopause. In this regard, it was assumed that factors of reproductive health, including age at menarche (AAM), age at natural menopause (ANM), age at first birth (AFB), age at first sexual intercourse (AFS), and pregnancy loss, could have an impact on women's risk of CSVD. However, two randomized controlled trial studies have failed to confirm the beneficial effect of daily estrogen treatments on brain structures (14, 15). Therefore, the association between women's reproductive traits and CSVD remains unclear.

Mendelian randomization (MR) is a robust technique to infer the causality between exposures and the risk of diseases by using genetic variants as instrumental variables with such exposures. Since genetic variants are randomly allocated at meiosis, confounding bias and reverse causation in observational studies could be avoided (16). The genetic regulations in AAM, ANM, AFB, AFS, and pregnancy loss have been recently highlighted by discoveries from large-scale genome-wide association studies (GWAS) leveraging millions of women of European ancestry. Meanwhile, various clinical and subclinical measures of CSVD have been revealed by publicly available GWAS, making it possible to explore the causal relationships between reproductive factors and CSVD. Notably, the development of functional neuroimaging technologies also presented new opportunities to investigate whether reproductive traits influence cerebral circulation and brain activation in ways that might impact CSVD in women.

In the present study, we conducted a two-sample MR to investigate the causal relationships between five women's reproductive traits and CSVD clinical (ICH by location or SVS) and subclinical measures (WMH). As supplementary outcome measures, two diffusion tensor imaging measures, fractional anisotropy (FA) and mean diffusivity (MD), were used as supplementary outcome measures as they capture early microstructural lesions of white matter attributing to CSVD. We aimed to gain a comprehensive understanding of the effects of reproductive factors on CSVD and to evaluate the results in terms of both clinical and neuroimaging findings.

## Materials and methods

### Study design

**Figure 1:**
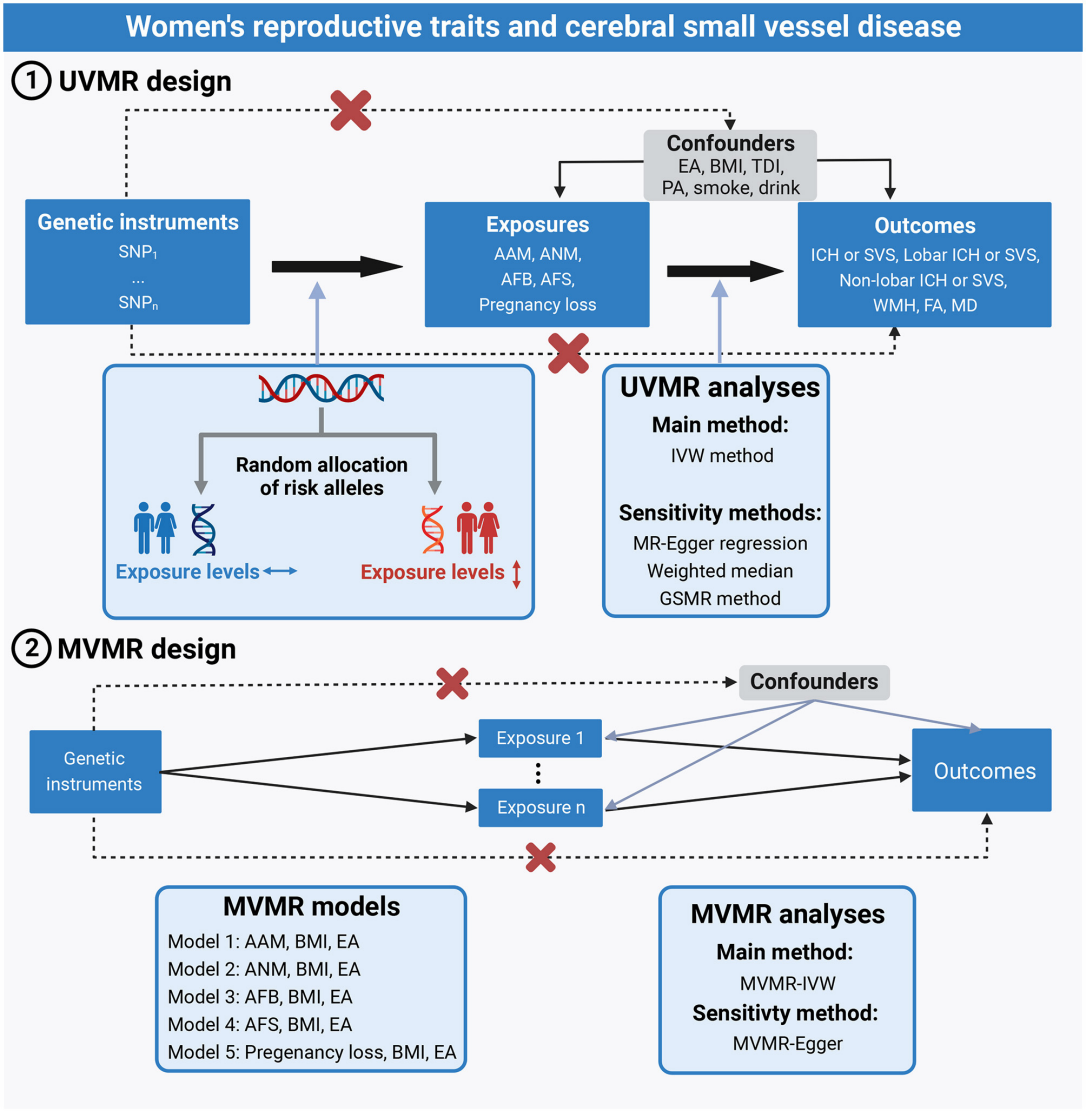
Assumptions and study design of the MR study of the associations between 5 women's reproductive traits and CSVD. AAM, age at menarche; AFB, age at first birth; AFS, age at first sexual intercourse; ANM, age at natural menopause; BMI, body mass index; CSVD, cerebral small-vessel disease; EA, educational attainment; GSMR, generalized summary-data-based Mendelian randomization; ICH, intracerebral hemorrhage; IVW, inverse-variance weighted; MVMR, multivariable Mendelian randomization; PA, physical activity; SVS, small-vessel ischemic stroke; TDI, Townsend deprivation index; UVMR, univariable Mendelian randomization.

A brief description of the two-sample MR designs is displayed in Figure 1. We performed two-sample univariable MR (UVMR) and multivariable MR (MVMR) to comprehensively explore the relationships between 5 women's reproductive traits on CSVD clinical measures (ICH by location or SVS) and neuroimaging features (WMH, FA, and MD). UVMR is based on the following three main assumptions: (1) the genetic variant selected as the instrumental variable is robustly associated with the exposure; (2) the genetic variant is not associated with confounders; and (3) the genetic variants affect the outcome only through the exposure, not other pathways (17). Compared with the assumptions of UVMR, the first assumption of MVMR was the genetic variants associated with one or more of the exposures, and other assumptions were consistent with UVMR (18). In this study, we first selected genetic variants for each woman's reproductive trait to infer the causality from each woman's reproductive traits to CSVD clinical outcomes and neuroimaging features using UVMR. Second, we integrated GWAS summary statistics and additional genetic variants on body mass index (BMI) and educational attainment (EA) and conducted the MVMR models to estimate the direct effect of reproductive factors on CSVD clinical outcomes and MRI markers of CSVD, controlling for the effect of BMI and EA.

### Data sources

In the current study, the exposures were women's reproductive traits, including AAM, ANM, AFB, AFS, and pregnancy loss. The study's outcomes were CSVD clinical outcomes (all location ICH or SVS, lobar ICH or SVS, and non-lobar ICH or SVS) and MRI markers of CSVD (WMH, FA, and MD). For each trait, summary-level data (effect estimates, standard errors, and *P*-values) were obtained from the newly published European GWAS (Table 1). As the calculated potential UK Biobank sample overlap between exposure and outcome GWAS was < 5% at maximum, we considered the risk of bias due to sample overlap minimal (19).

**Table 1:** Data sources used in the MR analyses for the current study.

**Phenotype**	**Participants included in the analysis**	**Ancestry**	**Type of trait**	**Adjustments**	**Sources**
**Exposures**					
AAM	329,345 females	European	Continuous	Age at study visit and other study-specific covariates	ReproGen, UK Biobank, 23andMe (20)
ANM	201,323 females	European	Continuous	Genetic PCs matrix	UK Biobank, BCAC Consortium, 1,000 Genomes imputed studies (21)
AFB	418,758 females	European	Continuous	Birth year of the respondent, its square, its cubic and top PCs	36 studies (22)
AFS	214,547 females	European	Continuous	Birth year of the respondent, its square, its cubic and top PCs	UK Biobank (22)
Pregnancy loss	191,252 females (60,565 cases and 130,687 controls)	European	Binary	Age, up to 20 genetic PCs	UK Biobank (http://www.nealelab.is/uk-biobank) (51)
Estradiol in sensitivity analysis	2,767 females	European	Continuous	Laboratory batch, study, age at blood draw, BMI, HRT use, and menopausal status	Sisters in Breast Screening study (52)
BioT in sensitivity analysis	188,507 females	European	Continuous	Age, genotyping batch, mins since blood draw, time of blood draw, menopause, and operation status	UK Biobank (53)
Progesterone in sensitivity analysis	3,501 females	European	Continuous	Sex, age, and population stratification	LIFE-Adult, and LIFE-Heart (54)
**Outcomes**					
WMH	18,381 individuals	European	Continuous	Age at MRI, sex, genotyping array, assessment center, 10 genetic PCs, and MRI head motion indicators	UK Biobank (25)
FA	17,663 individuals	European	Continuous	Age at MRI, sex, genotyping array, assessment center, 10 genetic PCs, and MRI head motion indicators	UK Biobank (25)
MD	17,467 individuals	European	Continuous	Age at MRI, sex, genotyping array, assessment center, 10 genetic PCs, and MRI head motion indicators	UK Biobank (25)
ICH or SVS	6,255 ICH or SVS cases and 233,058 controls	European	Binary	Age, sex, and first 4 genetic PCs	MEGASTROKE consortium, GOCHA, ISGC-EUR, GERFHS (24)
Lobar ICH or SVS	5,208 lobar ICH or SVS cases and 233,058 controls	European	Binary	Age, sex, and first 4 genetic PCs	MEGASTROKE consortium, GOCHA, ISGC-EUR, GERFHS (24)
Non-lobar ICH or SVS	5,468 non-lobar ICH or SVS cases and 233,058 controls	European	Binary	Age, sex, and first 4 genetic PCs	MEGASTROKE consortium, GOCHA, ISGC-EUR, GERFHS (24)

AAM, age at menarche; AFB, age at first birth; AFS, age at first sexual intercourse; ANM, age at natural menopause; FA, fractional anisotropy; GERFHS, genetic and environmental risk factors for hemorrhagic stroke; GOCHA, genetics of cerebral hemorrhage on anticoagulation; ICH, intracerebral hemorrhage; ISGC-EUR, European member sites contributing to the international stroke genetics consortium; MD, mean diffusivity; MRI, magnetic resonance imaging; PCs, principal components; SVS, small-vessel ischemic stroke; WMH, white matter hyperintensities.

### Women's reproductive traits

Genetic variants of AAM were obtained from a meta-analysis of GWAS, including 329,345 individuals of European ancestry from UK Biobank, 23andMe, and ReproGen consortium (20). Each dataset used an additive linear regression model to assess the associations between single nucleotide polymorphisms (SNPs) and AAM, adjusted for age at the study visit and other study-specific covariates. Then, the results of each dataset were combined using a meta-analysis of the inverse-variance-weighted meta-analysis method (20).

Genetic predictors of ANM were obtained from the up-to-date GWAS meta-analysis in ~200,000 women of European ancestry (21). ANM was defined by the age at the last naturally occurring menstrual period followed by at least 12 consecutive months of amenorrhea and derived from self-reported data in each study. Genetic associations between SNPs and ANM were obtained from the additive linear regression model adjusted for the genetic principal component matrix.

From 36 studies of European descent in total, Mills et al. reported the largest meta-analysis of GWAS, including 418,758 women for AFB (22). AFB was treated as a continuous measure, assessed for those who have ever given birth to a child. Genetic variants of AFS were also obtained from the largest GWAS, including 214,547 women of European ancestry from UK Biobank (22). AFS was treated as a continuous measure, with individuals considered eligible if they had given a valid answer, with ages lower than 12 years excluded, and was transformed by inverse rank-normal. Both genetic associations between SNPs and AFB or AFS were adjusted for the birth year of the respondent and its square, cubic, and top principal components (22).

Summary-level data for pregnancy loss was derived from the UK Biobank study (23). In UK Biobank, pregnancy loss was defined by the history of stillbirth, spontaneous miscarriage, or termination. We used the second round of Neale Lab's GWAS (http://www.nealelab.is/uk-biobank) in UK Biobank, which included 191,252 women (60,565 cases and 130,687 controls). Genetic associations were adjusted for 20 genetic principal components and age.

### CSVD clinical outcomes

The CSVD clinical outcomes were three cross-phenotype outcomes, including “all location ICH or SVS,” “lobar ICH or SVS,” and “non-lobar ICH or SVS.” The GWAS of the cross-phenotype outcomes were conducted by meta-analysis of the SVS GWAS from MEGASTROKE with ICH GWAS by location (all locations, lobar, and non-lobar) (24). Samples included 241,024 participants of European ancestry (6,255 ICH or SVS cases and 233,058 controls) from the MEGASTROKE consortium and three ICH datasets. Within each dataset, logistic regression with adjustments for age, sex, and the first four genetic principal components was performed for all location ICH, lobar ICH, and non-lobar ICH. Multi-trait analysis of GWAS (MTAG) was performed to integrate summary data across the two diseases and generate combined effect estimates.

### MRI markers of CSVD

Summary-level data for CSVD neuroimaging features (WMH, FA, and MD) were derived from GWAS of ~20,000 individuals from UK Biobank (25). Individuals diagnosed with stroke or other major central nervous system diseases that could be associated with WMH were excluded from the analyses. WMH is a radiological marker commonly used to identify CSVD, while FA and MD are measures of white matter microstructural integrity that are abnormal in CSVD. The WMH trait was log-transformed and normalized for brain volume. FA and MD were obtained from DTI images by performing principal component analysis on the FA and MD measures of each of the 48 different brain tracts analyzed. GWAS were performed using linear regression on WMH (*N* = 18,381), FA (*N* = 17,663), and MD (*N* = 17,467), with adjustments for sex and age at MRI, genotyping array, UK Biobank assessment center, the first 10 genetic principal components, and MRI head motion indicators as covariates (25).

### Selection of genetic instrumental variables

To satisfy the MR assumptions (Figure 1), all SNPs for UVMR and MVMR were strongly and independently (*R*^2^ < 0.001 within 10 Mb) predicted exposures from the published GWAS at genome-wide significance (*P* < 5 × 10^−8^). Since there were no genome-wide significant SNPs for pregnancy loss, we adopted a less stringent threshold of 5 × 10^−6^ to obtain more SNPs for pregnancy loss.

Using the publicly available GWAS summary data, we examined whether any of these SNPs were associated with confounders (BMI, EA, physical activity, Townsend deprivation index, smoking, and drinking) and outcomes at a *P*-value of 1 × 10^−5^ for UVMR. The associations of these SNPs with inverse-normally transformed BMI were obtained from a meta-analysis of GWAS in ~700,000 participants of European ancestry (26). The associations of these SNPs with alcoholic drinks per week and cigarettes smoked per day were obtained from a meta-analysis of GWAS in up to 1.2 million individuals of European ancestry (27). Summary-level data on the Townsend deprivation index were obtained from the MR-Base platform, including 462,464 individuals of European ancestry in UK Biobank. Summary-level data for physical activity were derived from a GWAS of 377,234 individuals from UK Biobank (28). Genetic associations with EA were obtained from the meta-analysis of GWAS on 3 million individuals of European ancestry contributed by a previous meta-analysis of 69 cohorts, 23andMe, and UK Biobank (29). Similar to UVMR, we also examined whether any of these SNPs were associated with confounders (physical activity, Townsend deprivation index, smoking, and drinking) and outcomes for MVMR.

Finally, we quantified the strength of SNPs for UVMR using mean *F*-statistics (30). Mean *F*-statistics of >10 suggested sufficient strength to ensure the validity of the SNPs for the exposures in UVMR. When two samples were overlapped, the conditional *F*-statistics to assess the strength of SNPs for MVMR were not calculated because the requisite pairwise covariances between SNP associations were determinable only using individual-level data (31).

### Statistical analysis

SNPs that were unavailable in the outcome datasets were replaced by proxies at *R*^2^ > 0.80 in LDlink (https://ldlink.nci.nih.gov/). After extracting and harmonizing the data, we performed UVMR to estimate the causal effect of women's reproductive traits on MRI markers of CSVD. In the main analysis, we calculated a Wald ratio estimate for each genetic variant and summarized the estimates using the inverse-variance-weighted (IVW) method. The IVW with the multiplicative random-effects method provides a concise estimation and accounts for potential heterogeneity among the Wald ratio estimates from SNPs (32). Thus, if there was heterogeneity, random-effects IVW models were applied; otherwise, the fixed-effect IVW model was applied. To assess the robustness of the findings, we also performed sensitivity analyses using methods with different assumptions about horizontal pleiotropy, including MR-Egger regression, weighted median, generalized summary-data-based MR (GSMR), and MR-pleiotropy residual sum and outlier (MR-PRESSO). An evaluation of instrumental variable pleiotropy was provided by MR-Egger analysis, with a non-zero intercept suggesting bias in the IVW estimate (33). The weighted median approach, which leveraged the weighted median estimator, was used to determine if several genetic variants were invalid or presented pleiotropy (34). The GSMR method accounts for both possible LD between SNPs and the sampling errors in the estimated effect sizes of the instruments on the exposures and excludes SNPs that show evidence of pleiotropic effects by the heterogeneity in dependent instrument outlier analysis (HEIDI-outlier test < 0.01) (35). MR-PRESSO uses the global test to detect horizontal pleiotropy and, if necessary, could correct for potential pleiotropic outliers *via* outlier removal. If corrected, the MR-PRESSO distortion test was used to test for statistically significant differences in the causal estimates before and after correction. If the *P*-values of both the global and distortion tests were < 0.05, which indicates the existence of horizontal pleiotropy, the outlier-adjusted causal estimates for relationships were presented; otherwise, the results before correction were presented (36). Heterogeneity in the IVW estimates was examined by the Cochran *Q*-test and *I*^2^-index.

We also performed MVMR to assess the direct effect of women's reproductive traits on MRI markers of CSVD, controlling for BMI and EA, because obesity and EA may play confounding roles in the pathway from women's reproductive traits to CSVD-related outcomes. For MVMR, we used an extension of the IVW-MR method, performing MVMR-IVW and selecting random effects or fixed effects based on heterogeneity as described in UVMR. The MVMR-Egger and MVMR-robust methods were used as sensitivity analyses. The MVMR-Egger method was proposed to correct for both measured and unmeasured pleiotropy (18). The MVMR-robust method was robust to pleiotropic SNPs by employing a robust regression to attenuate the influence of outlier IVs (37).

Since we employed sex-specific SNPs for women's reproductive traits, GWAS of CSVD-related outcomes were conducted in both men and women. We repeatedly performed UVMR and MVMR analyses utilizing sex-combined SNPs for AFB and AFS as sensitivity analyses to investigate the impact of the issue of sex heterogeneity of SNPs when conducting sex-specific two-sample MR studies. Since younger age at menarche and older age at menopause have been widely reported as risk factors for ovarian cancer, we performed a positive control study to examine the relationship between AAM and ANM with ovarian cancer and measure the efficacy of selected genetic instruments (20, 38). Data for epithelial ovarian cancer were derived from a meta-analysis of GWAS of 25,509 cases and 40,941 controls from the Ovarian Cancer Association Consortium (OCAC) (39). Considering the secretion of sex hormones throughout the process of female reproductive traits, we additionally conducted sensitivity MR analyses to investigate the associations between three women's sex hormones and CSVD. Detailed information for summary-level data of serum estradiol, bioavailable testosterone (BioT), and progesterone is described in Table 1. For estradiol with only one SNP available, we performed Wald ratio estimation as other MR methods require at least 2 SNPs.

The results of the effects of women's reproductive traits on CSVD clinical outcomes and CSVD neuroimaging features are presented as ORs (95% CIs) and βs (95% CIs), respectively. The Bonferroni method was used to correct for multiple testing, and therefore, we considered associations with *P*-values below 0.01 (0.05/5) as strong evidence of associations. The results with *P*-values between 0.01 and 0.05 were regarded as suggestive associations. All analyses were two-sided and conducted using TwoSampleMR (version 0.5.6), MVMR (version 0.3), and GSMR (version 1.0.9) packages in the R software (version 3.6.3). Reporting of the study follows the STROBE-MR statement.

## Results

### UVMR analyses of women's reproductive traits on MRI markers of CSVD

**Figure 2:**
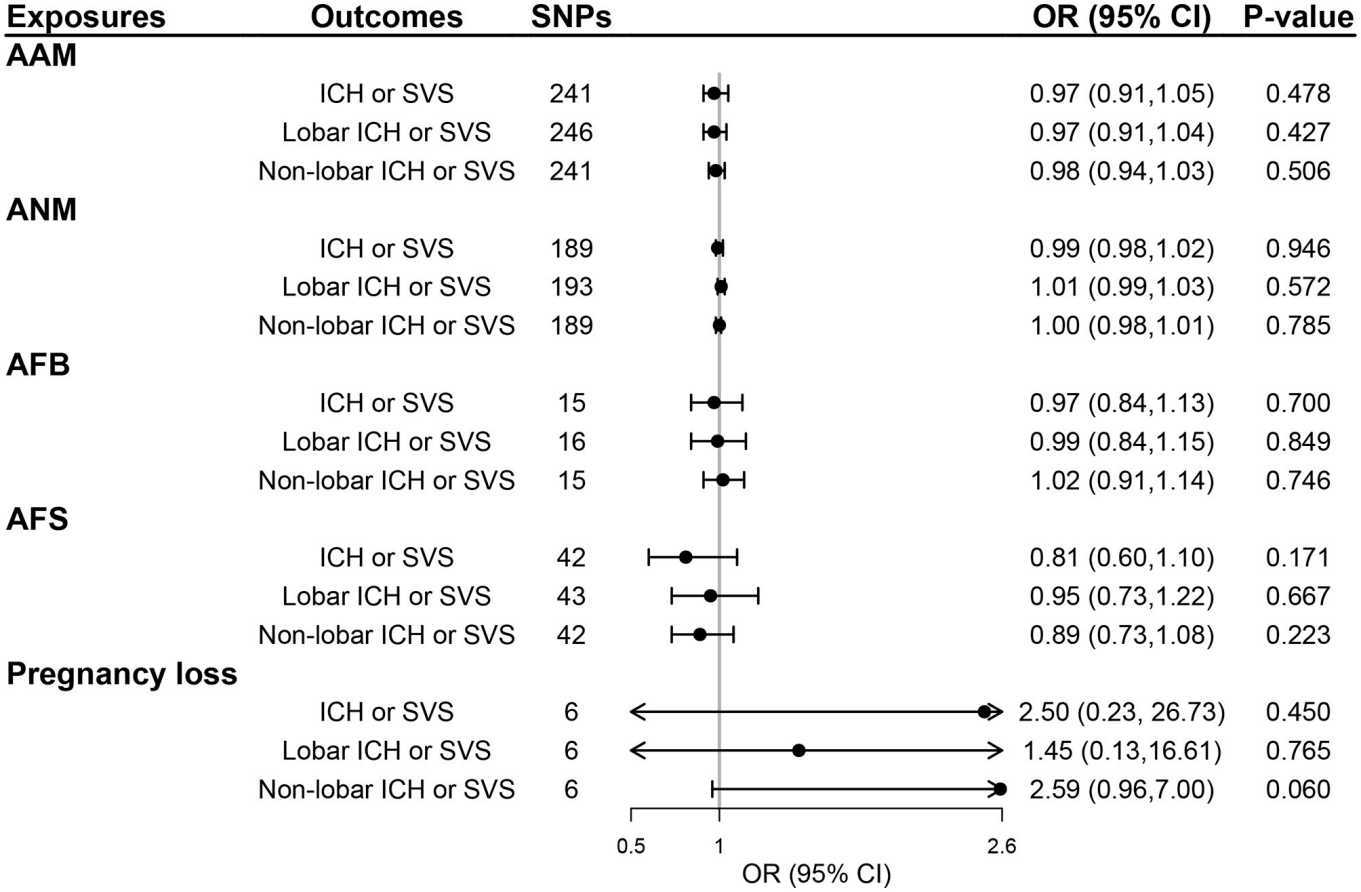
The effect of genetically determined women's reproductive traits on CSVD clinical outcomes using UVMR. AAM, age at menarche; AFB, age at first birth; AFS, age at first sexual intercourse; ANM, age at natural menopause; ICH, intracerebral hemorrhage; SVS, small-vessel ischemic stroke.

**Figure 3:**
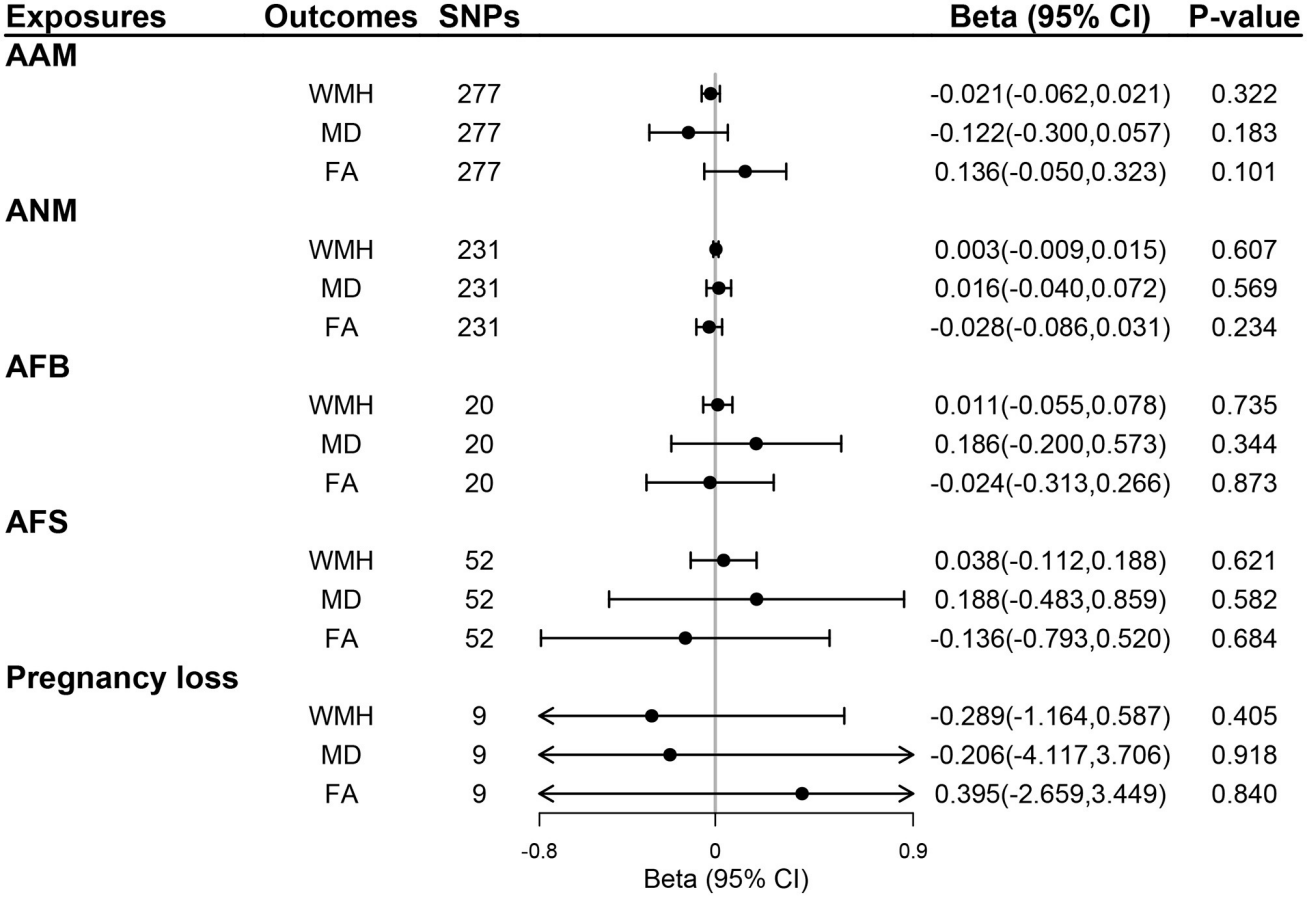
The effect of genetically determined women's reproductive traits on MRI markers of CSVD using UVMR. AAM, age at menarche; AFB, age at first birth; AFS, age at first sexual intercourse; ANM, age at natural menopause; FA, fractional anisotropy; MD, mean diffusivity; WMH, whiter matter hyperintensities.

We selected 319, 270, 25, 58, and 9 SNPs as genetic instruments for AAM, ANM, AFB, AFS, and pregnancy loss after linkage disequilibrium clumping and removing pleiotropic SNPs, respectively (Supplementary Tables S1–S5). The mean *F*-statistics for women's reproductive traits ranged from 22.78 to 92.57 (Supplementary Table S6), suggesting that the weak instrument bias was minimal. The primary IVW method did not show any evidence of the causal effect of five women's reproductive traits on the risk of ICH or SVS, lobar ICH or SVS, and non-lobar ICH or SVS (all *P* > 0.05; Figure 2) and also no evidence of the effect of genetically determined women's reproductive traits on WMH, FA, and MD (all *P* > 0.05; Figure 3). The results of MR-Egger regression and weighted median methods were consistent with the IVW method, and no horizontal pleiotropy was detected using the MR-Egger intercept test (Supplementary Table S7 *for MRI markers, S8 for CSVD clinical outcomes*). MR-PRESSO showed consistent results with the IVW method, and MR-PRESSO global test and distortion test also did not show any evidence of horizontal pleiotropy (Supplementary Tables S7, S8). GSMR also showed similar results after removing outliers detected by the HEIDI-outliers test (Supplementary Tables S7, S8). Although heterogeneity was detected in the UVMR of AAM and ANM on neuroimaging features of CSVD and UVMR of ANM on the risk of non-lobar ICH or SVS (all Q *P*-values < 0.05), we applied IVW with multiplicative random effects to mitigate the problem (Supplementary Tables S7, S8).

### MVMR analyses of women's reproductive traits on CSVD neuroimaging features

**Figure 4:**
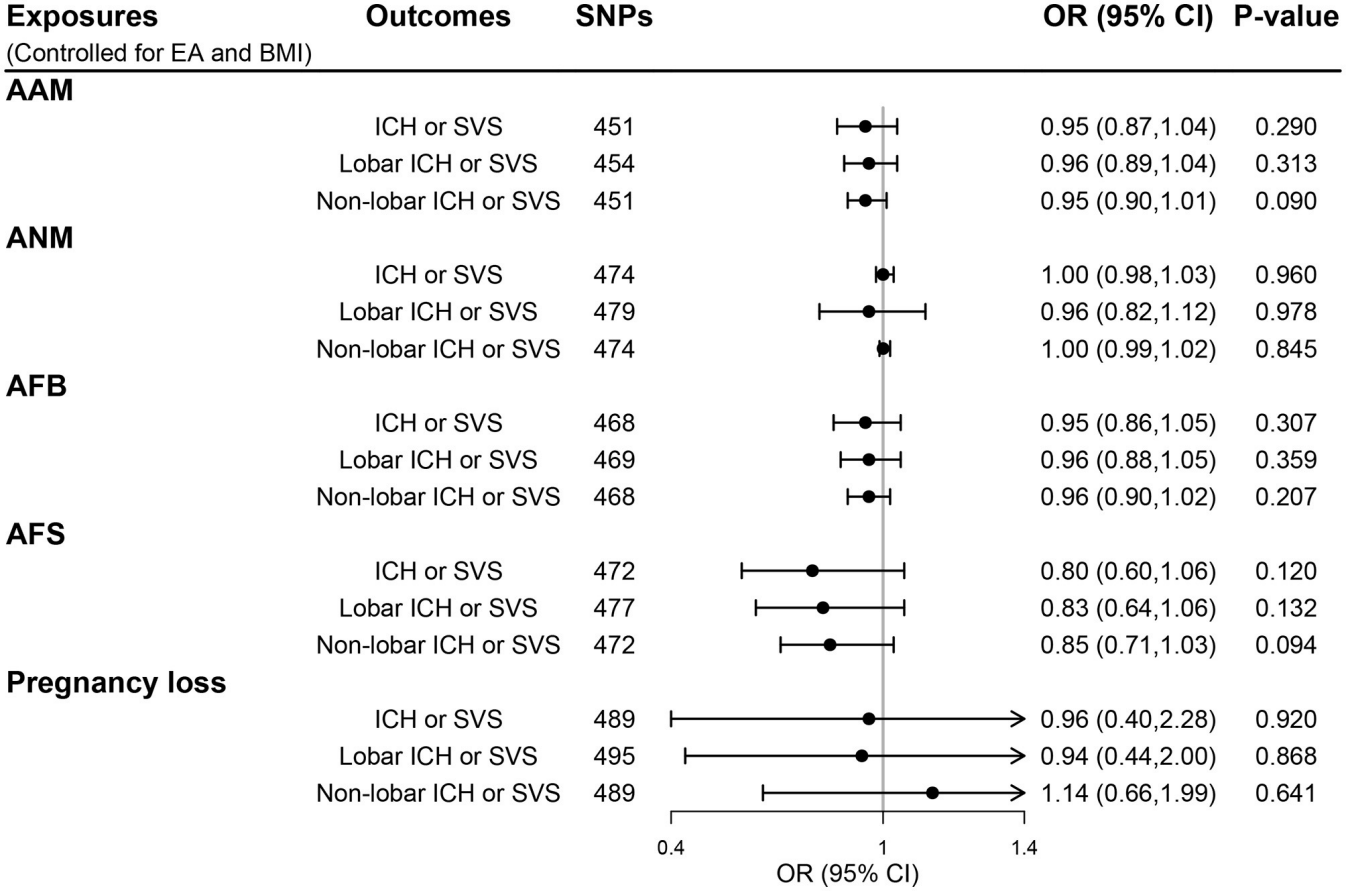
The direct effect of genetically determined women's reproductive traits on CSVD clinical outcomes using MVMR controlled for EA and BMI. AAM, age at menarche; AFB, age at first birth; AFS, age at first sexual intercourse; ANM, age at natural menopause; BMI, body mass index; EA, educational attainment; ICH, intracerebral hemorrhage; SVS, small-vessel ischemic stroke.

**Figure 5:**
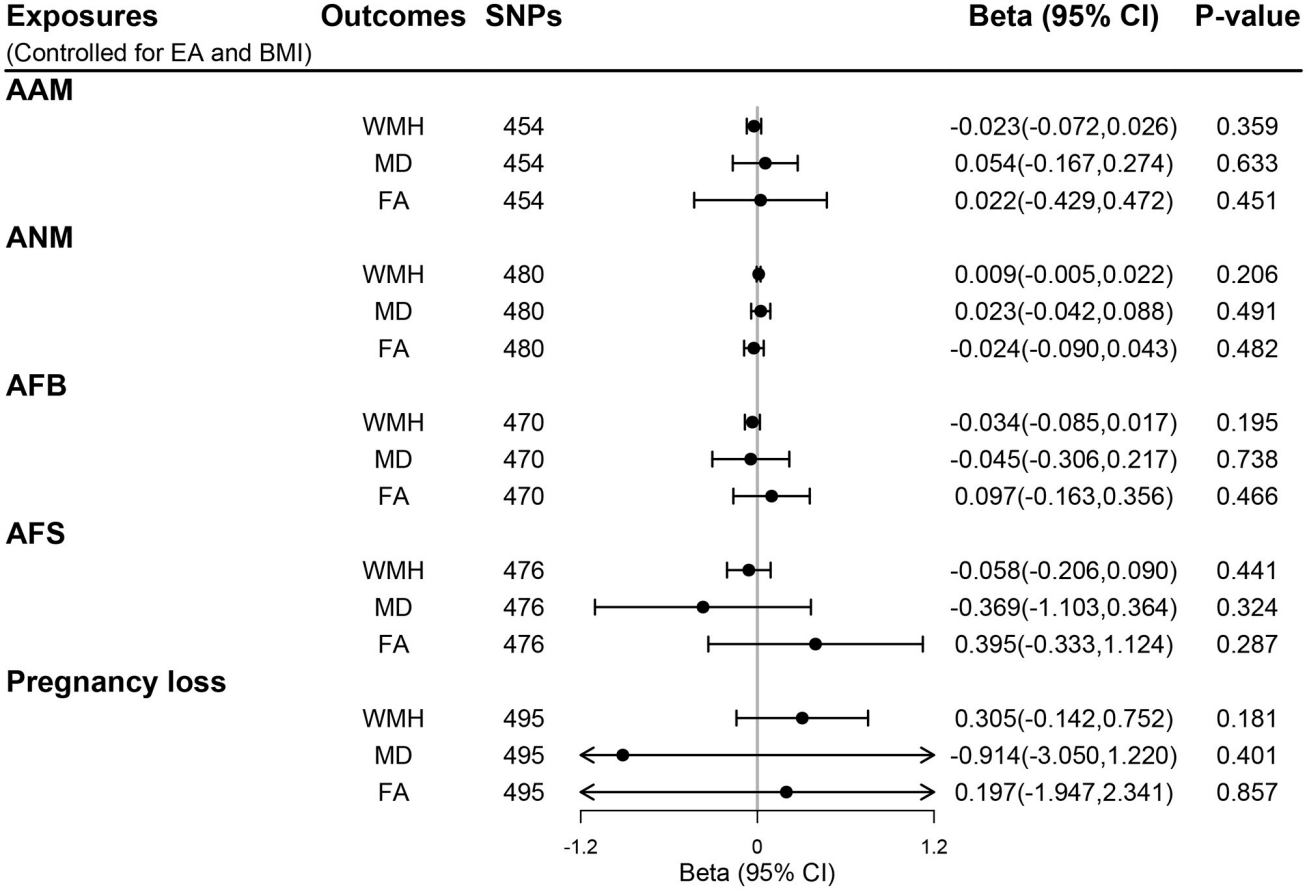
The direct effect of genetically determined women's reproductive traits on MRI markers of CSVD using MVMR controlled for EA and BMI. AAM, age at menarche; AFB, age at first birth; AFS, age at first sexual intercourse; ANM, age at natural menopause; BMI, body mass index; EA, educational attainment; FA, fractional anisotropy; MD, mean diffusivity; WMH, white matter hyperintensities.

Genetic instruments for BMI and EA are presented in Supplementary Tables S9, S10, respectively. Additional genome-wide significant genetic variants on EA and BMI were combined with each woman's reproductive trait in MVMR models, generating five MVMR models. After controlling for the effect of BMI and EA, there was no evidence for a direct effect of genetically predicted women's reproductive traits on CSVD clinical outcomes (all *P* > 0.05; Figure 4) and MRI markers of CSVD (all *P* > 0.05; Figure 5). Consistently, null findings were identified using the MVMR-Egger and MVMR-robust methods (Supplementary Table S11 *for MRI markers and* Supplementary Table S12 *for CSVD clinical outcomes*). We did not observe apparent signs of horizontal pleiotropy using the MVMR-Egger intercept test (all *P* for MVMR-Egger intercept >0.05; Supplementary Tables S11, S12).

### Sensitivity analysis

We also performed several sensitivity analyses to enhance the robustness of our results. First, we repeated UVMR and MVMR analyses using sex-combined genetic instruments for AFB and AFS to evaluate the impact of the sex heterogeneity of instruments on conducting sex-specific two-sample MR studies. Overall, the results were also similar to the primary analysis using women-specific SNPs for AFB and AFS. The UVMR and MVMR methods did not show any significant causal effect of AFB and AFS on CSVD clinical outcomes and neuroimaging features in the IVW method (Supplementary Tables S13, S14). MR-Egger and MVMR-Egger analyses did not show any evidence of horizontal pleiotropy for UVMR and MVMR, respectively (Supplementary Tables S13, S14). Second, positive control analysis demonstrated that genetic predisposition to older AAM was associated with a lower risk of ovarian cancer (OR [95% CI], 0.93 [0.88, 0.99]; *P* = 0.019), and older ANM was associated with a higher risk of ovarian cancer (1.02 [1.01, 1.04]; *P* < 0.001) in UVMR (Supplementary Table S15). MVMR controlling for EA and BMI also showed genetically determined AAM and ANM had inverse (0.96 [0.93, 0.99]; *P* = 0.042) and positive (1.03 [1.01, 1.06]; *P* = 0.001) direct effect on ovarian cancer, respectively. MR-Egger and MVMR-Egger intercept tests did not detect horizontal pleiotropy for UVMR and MVMR, respectively (Supplementary Table S15). The results further ensure the efficiency of the selected genetic instruments and our study design. Finally, we investigate the causal relationships between sex hormones and CSVD among women. The mean *F*-statistics for serum estradiol, progesterone, and BioT were all >10 (Supplementary Table S6), and the detailed information on genetic instruments for sex hormones is described in Supplementary Table S16. We observed no evidence of relationships between genetically predicted serum estradiol, BioT, and progesterone on MRI markers of CSVD (Supplementary Table S16), as well as CSVD clinical outcomes (Supplementary Table S17). Moreover, MR-Egger intercepts did not show any indication of directional pleiotropic effects for the analyses (all *P*-values for intercept >0.05, Supplementary Tables S17, S18).

## Discussion

In this study, we examined the causal relationship between five women's reproductive traits (AAM, ANM, AFB, AFS, and pregnancy loss) and the risk of CSVD in terms of its clinical manifestations and imaging features. We capitalized on the summary statistics of the largest GWAS conducted for these reproductive traits in European ancestry populations and constructed strong instruments using SNPs associated with exposures. However, we did not find convincing evidence supporting the causal effects of reproductive factors on CSVD using univariable MR analyses. The above results were robust to sensitivity analysis with other MR methods and did not show any evidence of horizontal pleiotropy, thus supporting the robustness of our findings. The putative positive causality was observed between AAM, ANM, and ovarian cancer, ensuring the efficacy of selected genetic instruments.

The current results from conventional epidemiological studies on this topic remain controversial, yet many studies suggest that estrogen may reduce the incidence and severity of cerebrovascular diseases. Some studies suggested that later menarche and earlier menopause, representing a shorter estrogen exposure period, could increase the chance of CSVD (40–42). However, we did not find any genetic evidence supporting the putative causal effects of female reproductive traits on CSVD clinical and neuroimage features, suggesting that estrogen exposure may not causally confer this protective effect on CSVD clinical and subclinical measurements. Notably, we observed a marginally significant effect of pregnancy loss on non-lobar ICH or SVS and a marginally significant effect of AAM on FA in UVMR. Nevertheless, the effects of pregnancy loss and AAM were largely attenuated in the MVMR analyses, suggesting no substantial evidence for the causality between the two female reproductive traits and CSVD.

Several reasons could underlie such a discrepancy, but there are essentially two main causes. First, reproductive factors are highly complex heterogeneous traits formed by both genetic and environmental factors, and the phenotypic variation of these traits cannot be completely captured by genetics alone. For example, AFB and AFS are mainly determined by psychosocial, cultural, and financial factors, while the genetic effects are unlikely to be independent of them. Meanwhile, the specific timing of reproductive events is mainly based on questionnaires. Imprecision in the measurement of these factors will lead to measurement bias, so we could not rule out the possibility of bias caused by the inaccurate recall in previous studies (13). Second, the incomplete control for confounding factors, including BMI and EA, that affect both the exposure and the outcome might contribute to the biased results of observational studies (43–46). For example, MR studies showed that a 1-year increase in AAM is associated with a 0.38 kg/m^2^ increase in adult BMI as well as a 0.14-year (53 days) increase in time spent in education (43, 44). An MR study showed that a genetic predisposition to higher BMI is associated with a higher burden of CSVD (47). Similarly, a large-scale prospective study demonstrated that low education was associated with more microbleeds and lower total brain volume (46). In this regard, our MVMR analysis controlled for the effects of BMI and education, and the negative results corroborated the main findings on a null association.

Our study has several strengths. No prior work has investigated the impacts of reproductive factors on CSVD due to a lack of studies following women through menarche, first sexual intercourse, first birth, menopause, and a history of miscarriage. For the first time, we incorporated five different reproductive traits reflecting estrogen exposure to explore the causal relationships between a wide range of women's reproductive traits and CSVD. The MR approach offered important benefits in terms of demonstrating causality while minimizing the possibility of reverse causation bias and the impact of environmental confounders. Notably, neuroimaging plays a pivotal role in the visualization of CSVD-related brain damage, and MRI biomarkers could also reflect poor cerebral blood flow regulation, a hallmark feature of CSVD. SVS and ICH are the two main clinical outcomes contributed by CSVD. Hence, this is an innovative attempt of considerable importance to understand whether women's reproductive traits contribute to changing the risk of CSVD from the perspective of MRI markers and clinical outcomes. Furthermore, compared with directly using a small sample size of available ICH-GWAS (48), the use of combined GWAS of ICH by location and SVS could improve statistical power. Methodologically, we additionally included ovarian cancer as a positive control outcome to detect unobserved confounding by verifying whether the well-established association is replicated in the observed sample. A positive control outcome can be defined as an outcome with a known non-null causal association with the exposure, which could be biased by a subset of unpredictable confounders for the exposure effects on the primary outcome (49). Our findings suggested a positive association of AAM and ANM with ovarian cancer, confirming that the results are valid and robust.

Some intrinsic limitations need to be considered when interpreting our findings. First, the summary-level data for women's reproductive traits only included women, whereas MRI markers of CSVD were tested in both men and women. Therefore, if the effects of the genetic variations differ between the two sexes, our results might be biased. To minimize the bias, we utilized SNPs for AFB and AFS from summary-level data comprising both men and women and found results that were similar to the main results. Second, there was a partial overlap between exposure samples and outcome samples, which may create bias among the results. In this regard, the strong strength of genetic instruments (all mean *F* > 10) and low degree of overlap (< 5%) suggested considerable bias would not be expected (19). Third, we performed the MR study under the linear assumption to evaluate the relationship between women's reproductive traits and CSVD neuroimaging features, while we cannot exclude a non-linear effect that was not captured by our study with the current availability of data. Future work on such topics may be focused on non-linear MR studies using individual-level data. Fourth, we cannot differentiate ICH-related and SVS-related women's reproductive traits, and to get a specific effect estimate on each manifestation, GWAS of cross-phenotype outcomes were used. However, our principal objective was to identify whether women's reproductive traits are associated with CSVD under maximal statistical power. Delicate differentiation of heterogenous CSVD phenotypes is emphasized less. Fifth, various pathologies can lead to increasing MRI signal intensity in the white matter, indicating that WMH is not diagnostically specific for CSVD. However, WMH is still a neuroimaging finding resulting from CSVD and is commonly used to identify CSVD. A recent study reported that a novel marker [the peak width of skeletonized mean diffusivity (PSMD)] is a more robust imaging marker for CSVD than WMH (50). It is worthwhile that investigating the causal association between women's reproductive traits and PSMD in the future may enhance the negative results of women's reproductive traits on CSVD. Finally, we only used summary-level data from participants of European descent, thus, our results cannot be generalized to other ethnicities.

## Conclusion

In summary, using both UVMR and MVMR, we found that there was no evidence supporting a causal effect of women's reproductive factors (including AAM, ANM, AFS, AFB, and pregnancy loss) on CSVD clinical and neuroimaging findings. These findings are a preliminary but important step toward determining the causal relationship between hormonal reproductive factors and CSVD in women. In addition, any potential cerebrovascular benefits of estrogen must be weighed against the known risk of ovarian cancer. The idea that estrogen has neuroprotective properties is a relatively new one and lacks evidentiary support; thus, understanding its exact role is critical for future CSVD prevention targeting high-risk women. Our knowledge on this topic is still insufficient, and future studies are warranted to investigate more specific physiological changes which may inform the researchers on the causal mechanisms involved in cerebral small-vessel disease progression.

## Data availability statement

The original contributions presented in the study are included in the article/Supplementary material, further inquiries can be directed to the corresponding author.

## Ethics statement

Ethical review and approval was not required for the study on human participants in accordance with the local legislation and institutional requirements. Written informed consent from the patients/participants or patients/participants' legal guardian/next of kin was not required to participate in this study in accordance with the national legislation and the institutional requirements.

## Author contributions

ZW, JL, and JZ contributed to the research questions and study design, data curation, methodology development, and conducted statistical analyses. ZW, JL, WW, and JZ helped validate and perform sensitivity analyses, interpreted the results and wrote the original draft of the manuscript, and helped review and edit the final draft of the manuscript. All authors read and approved the final manuscript.
